# Influenza1Read before the Pennsylvania State Veterinary Medical Association, March 6, 1900.

**Published:** 1900-06

**Authors:** Charles Williams

**Affiliations:** Philadelphia, Pa.


### INFLUENZA.[1]

By Charles Williams, V.M.D.,
PHILADELPHIA, PA.

Synonyms: Pink-eye, typhoid fever, epizoöty, bilious fever, and a number of names given to it by numerous writers on the Continent of Europe.

For our convenience we will allude to it as influenza.

*Definition.* Influenza is a contagious and infectious specific disease of the horse, ass, mule, and even of man, with alterations of the blood attended with more or less depression and stupefaction of brain and nervous system, and depression of the vital forces, with frequent complications of the important vascular organs, especially of the lungs, intestines, brain, and serous membranes, and the laminæ of the feet. One attack usually renders the animal immune to the disease for a longer or shorter period, but not always. This disease, under certain conditions of the atmosphere from unknown causes, may assume an epizoötic form or tendency, so that a whole neighborhood or even a country may be swept by its ravages in a few hours or days. The first description of this disease is said to have been made by Laurenteus Rusius, in A.D. 1301, when it spread over a considerable portion of Italy, causing great loss among the war horses of Rome. In 1648 it visited Germany in an epizoötic form, and spread from there to other parts of Europe. In 1711, under the name of "epidemica equorum," it followed the track of the great armies of Europe, causing great losses among

[1] Read before the Pennsylvania State Veterinary Medical Association, March 6, 1900.

the horses, while the rinderpest was scourging the cattle of the same regions. Thus we see that the awful business of war is and always has been productive of the greatest evils and of little good in comparison to the great devastations that follow in its wake.

In 1732 it is first mentioned in the British Isles, occurring in London and Southern countries of England, and was described by Gibson.

In 1758 Robert Whytt mentions the disease as devastating the horses in the north of Scotland. All through the eighteenth century it is mentioned in different parts of Europe.

In 1776 it first reached the shores of North America, but is not mentioned again until 1871 and 1872, when it swept the entire country from Canada south to Ohio, and then to the sea borders East and West. It now seems to be permanently rooted in our large cities, in such places as the shipping stables and markets where the green horses are handled and sold.

*Etiology*. As with many other contagious and infectious diseases one attack frequently renders the recovered subject immune for a variable length of time. The atmosphere is the most common carrier of the infection from the sick to the healthy animals, and may be carried to considerable distances. The contagion will remain in straw, bedding, and in the infected stable for some time. It is said water, even though running, may carry the infection. Dieckerhoff studied the disease in 1881 ; his experiments showed that the virus of influenza is excessively diffusible, and that it will spread rapidly and pass through ventilators or in any other way where a draught is created. He explains in this way the very irregular manner in which the disease attacks the horses in a stable. With all due respect to his opinions in the matter, your essayist holds to the opinion that it is the susceptible patient who first contracts the disease ; the plethoric animal, or one that has rapidly gained flesh and fat, whose liver is possibly torpid, the bile ducts possibly a little clogged, etc., as horses that are fitted for market with little exercise, and again old, highly-fed workers that have not had any grain feed but the concentrated feeds, and hay year in and year out, these are the ones to take the disease, and these are the ones that take on the bilious type, and are often subject to such serious complications. A perfectly healthy animal, one in which every organ is performing its function properly, will be able (in my opinion) to throw off the infection and thus escape, even though in direct proximity to the disease.

*Incubation*. From five to seven days after a susceptible horse has been exposed to the disease he may appear in usual good health, when all at once, almost as though he were struck, he may show the signs of the disease, which may be at once very pronounced and cause much alarm, or it may start in a very mild form, such as a faint cough, animal not quite finishing feed, a slight elevation of temperature, with little or no alteration of pulse, which gradually progresses to the highest point, say 105° or 106°, and finally the pulse in sympathy running up to 56 or 60 or even 80 per minute ; the latter is a cause of much uneasiness to the writer, especially about the fourth or fifth day. In the first case the fever may assume the highest form, the animal dull and dejected, not noticing passing objects. He frequently has rigors in croup and flank muscles. Yawning and grinding of the teeth, the latter symptoms showing there is severe gastric and hepatic derangement, and will indicate (as Huidekoper truly says) that a severe attack may be expected. The eyes become puffy, and tears run down over the cheeks. Animal seems to show great fatigue, frequently shifting from one leg to the other, the changing often attended with a cracking in the joints and tendons; if forced to move the animal will stagger and drag the limbs. The conjunctiva may be a bright pink or a deep saffron color, as also the lips. The appetite is variable. Some horses will keep on feeding through the attack when not complicated with bilious trouble, but when the latter is the permanent symptom the appetite is almost always wanting.

The limbs may begin to swell at the outset and become very much tumefied (usually in the simple and uncomplicated cases), or they may remain perfectly clean till after the subsidence of the former, when they will begin to swell until they reach the penis and under surface of the body. Usually after the temperature reaches its maximum (which may be at once or when the patient is first seen, or it may not be for a day or two), it remains stationary for a period of three to four days without much variation, when it may abate either gradually, say one or two degrees a day, or rapidly, almost as fast as it appeared, or in a few cases act intermittently for several days.

*Course and Complication.* The average duration of the disease is from six to ten days, in serious cases two to three weeks, and in benign cases three to six days. The fever subsides, the animal begins to take notice and start eating, the limbs may or may not become œdematous, and finally the mucous membranes clear up and the patient regains his strength and flesh.

The complications are : 1. Pneumonia with or without pleurisy.

2. Cardiac asthma. 3. Cerebritis. . 4. Colliquative diarrhœa with death. 5. Laminitis.

*Diagnosis.* The diagnosis consists of or is based on continued high fever, great depression, color of mucous membranes with or without conjunctivitis, swollen legs.

*Prognosis.* According to Huidekoper, influenza is considered to be a very serious disease, many horses dying even with the best treatment, and is more fatal to the well bred and young. The disease seems to occur in a more serious form in well bred than in lymphatic animals; the fatalities vary in different epizoötics, and the disease also seems to expend its force, so to speak, and the mortality is less toward the last than at the beginning of the break. The great danger seems to be in the dreaded complications, and just so sure as there is one case of pneumonia in a stable just so surely will the following cases be complicated with this disease, which usually makes its appearance about the fourth or fifth day. Other writers—Robertson, Friedberger and Fröhner, and Williams—say or consider it as a very mild or slightly serious disease. While I think these writers are only partially right, for, like the grip in the human race, it is much more mild at one time than another, and as I have before stated it is greatly dependent on the condition of the animal contracting the disease.

The pathology of the disease I will leave, and refer you to the able Continental writers.

*Treatment.* The treatment I will divide into two classes: 1. Curative. 2. Preventive.

The curative treatment is to guide the animal through the regular course of the disease and watch for and combat or treat any complications as they threaten to arise.

And I now come to the important part of my essay, namely, the preventive treatment or inoculation for immunizing our animals, especially in an outbreak of the disease. H. K. Mulford & Co., Philadelphia, are, as you all know, manufacturing chemists, and among their numerous products of different antitoxins have for some time produced an antitoxin which when injected into a child suffering with that dread disease diphtheria was capable of arresting the disease to a marked degree; it was also found that by injecting it into fowls suffering with roup they rapidly recovered, and it is thought by some who have tried it that it has been efficacious in distemper in dogs. Well, this then led to some experiment or at least some clinical testing of the serum as a preventive and curative treatment among the recently purchased horses coming from the West and passing through our West Philadelphia stables, as you all know a large percentage of these horses not only get very sick and some die and many more only recover after some time of nursing and care. It was, therefore, decided to test the serum to some extent in this line, and my friend, Dr. George Fuller, was given an opportunity to test it, and the results of his use of said serum seem to show that a green horse or an animal exposed to the infection of influenza, if not already sick, if injected with 20 c.c. of what Mulford calls his distemper antitoxin will not contract the disease in nearly every case, but if it should contract the disease it usually runs the prescribed course, but in a much milder form than others not inoculated, and usually if inoculated at the start, say when the temperature has risen to 102° F., the horse will usually recover at once, and the fever disappear. In the beginning of December or in the latter part of November, 1899, a single horse in the livery stable where I board my horses became affected with this disease, and gradually contracted more or less of a bronchitis, but otherwise ran a regular course and recovered. He had been well about a week or ten days when my attention was called to a black gelding, ten or twelve years old, which had been purchased about a month before (not green) in poor condition, which was a good feeder and laid on flesh rapidly; he became affected, after a while showed signs of pneumonia, and died of heart failure. His mate then became affected, and the disease seemed to have set in in earnest, till we had six or seven affected at one time, and they seemed to be taken down at the rate of one or two a day. I had learned of antitoxin as a curative agent, and began its use at once, and called Dr. Fuller on the 'phone, and asked his experience, and was advised to inoculate the well horses in the stable to stop the spread of the disease, which I did at once, and there were no more cases, with the exception of one which did not come on till a week or so after, and he had it only in the regulation form, and yielded without complications, while all the horses but one that contracted it after No. 2 had a complication of pneumonia. There was a second outbreak in another stable where I attended at the same time as the foregoing, and I inoculated somewhere about twenty head in it, and Dr. Fuller some others. Several of these cases started with a temperature of about 102°, but all dropped back to normal, went on working, and there was no more spread of the disease. I did some others—I might mention a green horse—which I found had a temperature of 102° F. in a small stable, with its mate; both were inoculated and recovered at once and went on working. In all I believe I inoculated about forty-seven horses in seven days, and in the lot there was only one that contracted the disease. The peculiarity of this outbreak in our stable was that many of the cases started with slight elevation of temperature, and gradually increased or remained with 2° or 3° of fever till four or five days, when pneumonia would set in and the temperature run up to 105.6°. From the above I am led to believe that as a preventive the serum seems to render an animal immune. As a curative agent I fear it will give little results, as the germs multiply so rapidly and produce so much toxin that we cannot get enough serum into the system to counteract its ravages.

